# Dataset on multichannel connectivity and video transmission carried on commercial 3G/4G networks in southern Sweden

**DOI:** 10.1016/j.dib.2019.104192

**Published:** 2019-06-25

**Authors:** Anders Johansson, Magnus Esbjörnsson, Per Nordqvist, Stig Wiinberg, Roger Andersson, Bodil Ivarsson, Bengt Eksund, Sebastian Möller

**Affiliations:** a Department of Clinical Sciences, Lund University, Sweden; b Office of Medical Service, Region Skåne, Sweden; c Department of Internal Medicine, Hässleholm Hospital, Sweden; d Department of Cardiothoracic Surgery, Lund University and Skåne University Hospital, Sweden

**Keywords:** Videotransmission, Telemedicine, Real-world, Cellular networks

## Abstract

In this data article, we report real-world data on multichannel connectivity and videotransmission carried on commercial 3G/4G networks in the region of Skåne, southern Sweden. The data reported here complement the research article “Technical feasibility and ambulance nurses’ view of a digital telemedicine system in pre-hospital stroke care – A pilot study” (1).

The dataset was originally collected as part of a project aimed to test in a clinical setting the quality and usefulness of a linked image and sound transmission in the prehospital assessment of patients with suspected stroke. The project built on previous studies indicating that using high-quality telemedicine in stroke cases is feasible and has already impacted local stroke care Schwamm et al., 2009. In addition, studies support the hypothesis that stroke telemedicine consultations, compared with telephone-only, result in more accurate decision-making Demaerschalk et al., 2012.

Cellular networks for 3/4G networks have been greatly improved, a prerequisite for the use of these networks for e. g. medical applications. However, connectivity maps for planning purposes are usually based on theoretical values that do not consider smaller features of the terrain such as large trees, hills, rocks etc. and that may interfere with connectivity.

To leverage several networks, multichannel devices have been developed that split the original transmission onto several independent channels and recombine the transmission on the receiver side. This setup allows to increase the available bandwidth and introduces at the same time an element of redundancy, provided that several providers with independent networks are used.

**Table:** Specifications table

Subject area	Video transmission over cellular networks
More specific subject area	Connectivity and ability to transmit large amounts of data, e.g. real-time videostreams
Type of data	Figures, tables
How data was acquired	A test vehicle, equipped with 2 high-definition video cameras (Axis F1035, AXIS F44) and a sound unit (AXIS A8105) that were connected via a local TCP/IP network to a multi-channel modem (Viprinet 512N). The modem communicated with a hospital base station (Viprinet Multichannel VPN Hub 1020). While traveling, connectivity data were recorded on the sender side (vehicle) using Viprinet software, while the corresponding videostreams were recorded and evaluated manually on the receiver side (hospital); interruptions of videosignals from individual cameras were annotated and mapped onto the connectivity data.
Data format	Tables and figures with summary and annotations of raw data
Experimental factors	Real life transmission of videosignals from moving vehicle travelling through both city and rural areas
Experimental features	A test vehicle was equipped with two videocameras that were connected via an internal network to a multichannelmodem (Viprinet) that divided the stream onto four parallel and redundant channels. The individual streams were carried on commercial cellular networks (3/4G) from different providers and via a hub (Viprinet) on the receiver side re-combined to one single stream. For details compare methods section.
Data source location	Skåne county, Sweden (see Fig 1)
Data accessibility	The reported data is within this article
Related research article	A Johansson; M Esbjörnsson; P Nordqvist; S Wiinberg; R Andersson; B Ivarsson, S Möller *“Technical Feasibility and Ambulance Nurses' View of a Digital Telemedicine system in Pre-hospital Stroke Care – A Pilot Study”* International Emergency Nursing (2019), https://doi.org/10.1016/j.ienj.2019.03.008[1]

**Table:** 

**Value of the data** • First publically available real-world data on multichannel connectivity and videotransmission carried on commercial 3G/4G networks in southern Sweden, supplementing model-based data. • These data may be of value to anyone designing studies and technical solutions in projects that need reliable transmission of large quantities of data from vehicles that move through less densely populated areas. • These data may be of value in the development of test protocols to identify areas with limited 3/4G coverage.

## 1 Data

In this article, we present maps, tables and descriptive text summarizing real world data on the use of 3/4G multichannel devices in this particular region of southern Sweden.

**Fig. 1:**
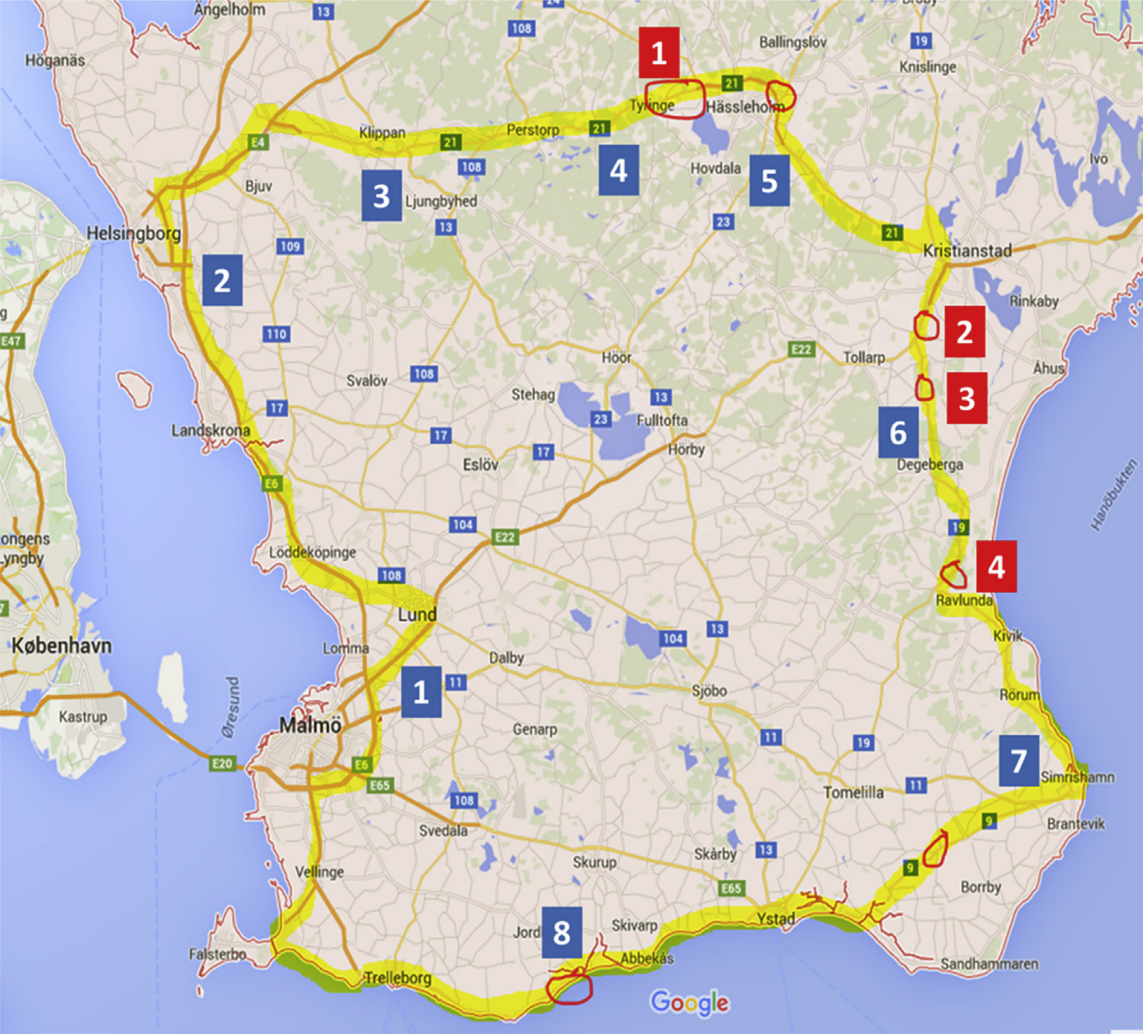
Map of the test area in southern Sweden.

Fig. 1: A graphical map of the test area. The map includes the route the test-vehicle took (yellow mark). In addition, areas of interruptions in the videostreams (red circles), with the longer interruptions numbered in red from 1 to 4 (Table 1) as well as corresponding readings of signal strength (Table 2) are annotated.

**Table 1:** Interruptions of video transmission ordered by the time of their occurrence.

Time Start	Time End	Duration	Map Point (red)
11:25:30	11:26:19	00:00:49^a^	1
13:00:56	13:00:58	00:00:02	
13:29:42	13:29:45	00:00:03	
13:34:13	13:34:52	00:00:39	2
13:41:31	13:42:08	00:00:37	3
13:54:29	13:54:46	00:00:17	4
14:49:16	14:49:18	00:00:02	
16:26:48	16:26:50	00:00:02	

a Both cameras affected.

**Table 2:** Examples of signal strength readings from modem software.

Time	Provider 1%	Provider 2%	Provider 3%	Provider 4%	Map Point (blue)
08:47	>90	>90	>90	>90	1
10:08	>90	>90	>90	>90	2
11:10	<50	>90	>90	>90	3
11:30	>90	>90	>90	>90	4
11:47	<90	>90	>50	>90	5
13:45	>90	<50	>90	>90	6
14:27	50–80	50–80	50–80	50–80	7
16:43	50	50	0	50	8

Table 1: The table lists time and duration of the larger interruptions in the received videostream. Note that only the first affected both cameras. Map Points refer to Fig. 1.

Table 2: Examples of signal strength readings obtained from the Viprinet modems software. Map Points refer to Fig. 1.

Areas of occasional interruptions are generally located in rural environment with obstacles such as hills and wood. They also correlate well to anecdotal reports from ambulance personnel familiar with these areas and experience from the clinical evaluation [1]. With the exception of the first interruption only one camera was affected, indicating that sufficient bandwidth for supporting at least one videostream was available during most of the duration of the test.

Note that signal strength was not recorded continuously, but at certain points of interest. During the course of the route, substantial variation in signal strength was noted for all providers. However, these fluctuations in individual channels did in general not overlap to the extent that no useable total signal (i.e. ≤ 50% in all channels) was received, which correlates well with the relatively low number of interruptions of the stream.

## 2 Experimental design, materials, and methods

We developed and evaluated a telemedicine application [1] that provides video and voice transmission for medically intended use between an ambulance-mounted camera/audio system and computers at the hospital. The project built on previous studies that indicated that the use of high-quality telemedicine is feasible and has already impacted lokal stroke care [2]. In addition, studies support the hypothesis that stroke telemedicne consultations, compared with telephone-only voice communication, result in more accurate decision-making [3]. In brief, the system consists of a test vehicle equipped with 2 high-definition video cameras (Axis F1035, AXIS F44) and a sound unit (AXIS A8105) that are connected via a local TCP/IP network to a multi-channel modem (Viprinet 512N). The modem simultaneously uses four commercial cellular connections to communicate with a hospital base station (Viprinet Multichannel VPN Hub 1020) within the hospital's network [4].

The data reported here were collected during the development phase of the system. The test vehicle travelled on a weekday during 9 hours (8am to 5pm) a total distance of approximately 400km with varying speed. Both densely populated areas (mostly on the western side of the area) and more rural areas in the countryside with a varying geography ranging from coastal areas and plains with agriculture to dense woods and steep hills were covered (Fig. 1).

For our test, signal strength was recorded as % of maximal strength using direct readings from the Viprinet modem's software. The videostreams were recorded and any interruptions on the cameras annotated. By manually mapping interruptions in videotransmission onto the connectivity data, areas of possibly insufficient coverage were identified and could be used in further risk-analysis of the system.

## References

[1] Johansson A., Esbjörnsson M., Nordqvist P., Wiinberg S., Andersson R., Ivarsson B., Möller S. (2019). Technical feasibility and ambulance nurses' view of a digital telemedicine system in pre-hospital stroke care – a pilot study. Int. Emerg. Nurs..

[2] Schwamm L.H., Holloway R.G., Amarenco P. (2009). A review of the evidence for the use of telemedicine within stroke systems of care. Stroke.

[3] Demaerschalk B.M., Raman R., Ernstrom K., Meyer B.C. (2012). Efficacy of telemedicine for stroke: pooled analysis of the stroke team remote evaluation using a digital observation camera (STRokE DOC) and STRokE DOC Arizona telestroke trials. Telemed. J. E. Health.

[4] Concept of Viprinet Connections (Providers Webpage): https://www.viprinet.com/en/technology/how-viprinet-works https://www.viprinet.com/en/solutions/industries/broadband-internet-vehicles (Accessed 2019-*05*-*02*).

